# On validly published names, correct names, and changes in the nomenclature of phyla and genera of prokaryotes: a guide for the perplexed

**DOI:** 10.1038/s41522-024-00494-9

**Published:** 2024-03-11

**Authors:** Aharon Oren

**Affiliations:** Department of Plant and Environmental Sciences, The Institute of Life Sciences, The Edmond J. Safra Campus, The Hebrew University of Jerusalem, Jerusalem, Israel

**Keywords:** Bacteriology, Bacteria

## Abstract

The nomenclature of prokaryotes is regulated by the rules of the International Code of Nomenclature of Prokaryotes. In view of inconsistencies in the use of names of many prokaryotic taxa and confusion caused by recent nomenclature changes, this essay presents an overview of how to use correct names of taxa. It includes information on validly published names of prokaryotic phyla, the way to deal with names of species that were transferred to newly created genera, and the nomenclature of uncultivated Candidatus taxa. It also provides recommendations for databases that provide reliable nomenclature information.

## Introduction

“Microbiologists who have occasion to use the scientific names of the micro-organisms with which they deal generally prefer to use *correct* names and use them *correctly*.” This opens the foreword to the first edition (1958) of the *International Code of Nomenclature of Bacteria and Viruses*^1^, the precursor of today’s *International Code of Nomenclature of Prokaryotes* (ICNP)^2^, the document that contains the internationally accepted rules that regulate the naming of prokaryotic taxa.

The editors of *NJP Biofilms and Microbiomes* have noticed inconsistencies in the use of names of prokaryotic taxa in the journal, and many colleagues are confused by a number of recent changes in the naming of taxa. Therefore, I here present a brief overview on how the nomenclature of prokaryotes is regulated and how to use correct names of taxa and how to use them correctly. I thank the editors of the journal for giving me the opportunity to explain some of the rules. Many names of phyla and of genera of prokaryotes were changed recently, and the status of such names is not clear to all.

The rules by which prokaryotes are named, as fixed in the ICNP^2^, are determined by the International Committee on Systematics of Prokaryotes (ICSP; the-icsp.org), a committee of representatives from the national microbiological societies and co-opted members. The ICSP is part of the Bacteriology and Applied Microbiology Division of the International Union of Microbiological Societies. The ICSP also supervises the publishing of the *International Journal of Systematic and Evolutionary Microbiology* (IJSEM). For valid publication, a name must be cited in the IJSEM (before 2000, the *International Journal of Systematic Bacteriology*) and must conform to the requirements laid down in the ICNP, or included in one of the Validation Lists published bimonthly in the IJSEM. Valid publication requires the designation of a nomenclatural type. In the case of species or subspecies the culture collections numbers of at least two publicly accessible service collections in different countries where a subculture of the type strain has been deposited must be indicated. The ICSP Judicial Commission issues Opinions concerning matters related to the interpretation of the ICNP. The ICNP regulates nomenclature (giving names to taxa), but it does not provide rules and guidelines on classification of prokaryotes (the arrangement of taxa into groups). This is clearly stated in Principle 1(4): “Nothing in this Code may restrict the freedom of taxonomic thought or action”.

Rule 27 of the ICNP explains the requirements for the valid publication of names. Public databases that are widely used by microbiologists as sources of information about prokaryotic taxa do not always use validly published names. A commendable effort was recently made to validate a large number of names of higher taxa (rank of family and higher) found in the Genome Taxonomy Database^3^ (GTDB; gtdb.ecogenomic.org), first by providing effective publications of the names^4^, followed by submission of 224 names for validation in a Validation List in the IJSEM^5^.

In 2021, the members of the ICSP accepted a proposal to include the rank of phylum in the ICNP^6^. The emended Rule 8 states that the name of a phylum is formed by the addition of the suffix -*ota* to the stem of the name of the designated type genus. This opened the way to the valid publication of the first 42 phylum names^7^. For example, the names *Pseudomonadota*, *Bacillota*, *Actinomycetota*, *and Bacteroidota*, with type species *Pseudomonas* Migula 1894 (Approved Lists 1980), *Bacillus* Cohn 1872 (Approved Lists 1980), *Actinomyces* Harz 1877 (Approved Lists 1980), and *Bacteroides* Castellani and Chalmers 1919 (Approved Lists 1980), were introduced for the bacterial taxa formerly known as *Proteobacteria*, *Firmicutes*, *Actinobacteria*, and *Bacteroidetes*, respectively. The archaeal phyla formerly known as *Euryarchaeota*, *Crenarchaeota* and *Thaumarchaeota* now also have validly published names: *Methanobacteriota*, *Thermoproteota*, and *Nitrososphaerota*, with type genera *Methanobacterium* Kluyver and van Niel 1936 (Approved Lists 1980), *Thermoproteus* Zillig and Stetter 1982, and *Nitrososphaera* Stieglmeier et al. 2014, respectively. The older phylum names should no longer be used, as only phyla names based on the stem of a designated type genus and the –*ota* ending are validly published under the rules of the ICNP. As of 3 February 2024, names of 49 phyla were validly published. These are presented in Table 1, together with the older names that were never validly published.

**Table 1:** The validly published names of prokaryotic phyla as of 3 February 2024, with the names of their type genera and names of older, effectively but not validly published names

Validly published name	Type genus	Older, not validly published names
*Abditibacterota*	*Abditibacter*	
*Acidobacteriota*	*Acidobacterium*	*Acidobacteria*
Actinomycetota	*Actinomyces*	*Actinobacteria*
*Aquificota*	*Aquifex*	*Aquificae*
*Armatimonadota*	*Armatimonas*	*-*
*Atribacterota*	*Atribacter*	*-*
*Bacillota*	*Bacillus*	*Firmicutes*, *Firmacutes*
*Bacteroidota*	*Bacteroides*	*Bacteroidetes*
*Balneolota*	*Balneola*	*Balneolaeota*
*Bdellovibrionota*	*Bdellovibrio*	-
*Caldisericota*	*Caldiserica*	*Caldisericia*
*Calditrichota*	*Caldithrix*	*Calditrichaeota*
*Campylobacterota*	*Campylobacter*	*Epsilonbacteraeota*
*Chlamydiota*	*Chlamydia*	*Chlamydiae*
*Chlorobiota*	*Chlorobium*	*Chlorobi*
*Chloroflexota*	*Chloroflexus*	*Chloroflexi*
*Chryseogenota*	*Chryseogenes*	*Chryseogenetes*
*Coprothermobacterota*	*Coprothermobacter*	-
*Cyanobacteriota*	*Cyanobacterium*	-
*Deferribacterota*	*Deferribacter*	*Deferribacteres*
*Deinococcota*	*Deinococcus*	*Deinococcus-Thermus*
*Desulfobacterota*	*Desulfobacter*	*-*
*Dictyoglomerota*	*Dictyoglomus*	*Dictyoglomi*
*Elusimicrobiota*	*Elusimicrobium*	*Elusimicrobia*
*Fibrobacterota*	*Fibrobacter*	*Fibrobacteres*
*Fusobacteriota*	*Fusobacterium*	*Fusobacteria*
*Gemmatimonadota*	*Gemmatimonas*	*Gemmatimonadetes*
*Ignavibacteriota*	*Ignavibacterium*	*Ignavibacteriae*
*Kiritimatiellota*	*Kiritimatiella*	*Kiritimatiellaeota*
*Lentisphaerota*	*Lentisphaera*	*Lentisphaerae*
*Methanobacteriota*	*Methanobacterium*	*Euryarchaeota*
*Microcaldota*	*Microcaldus*	-
*Mycoplasmatota*	*Mycoplasma*	*Tenericutes*
*Myxococcota*	*Myxococcus*	*-*
*Nanobdellota*	*Nanobdella*	-
*Nitrososphaerota*	*Nitrososphaera*	*Thaumarchaeota*
*Nitrospinota*	*Nitrospina*	*Nitrospinae*
*Nitrospirota*	*Nitrospira*	*Nitrospirae*
*Planctomycetota*	*Planctomyces*	*Planctomycetes*
*Pseudomonadota*	*Pseudomonas*	*Proteobacteria*
*Rhodothermota*	*Rhodothermus*	*Rhodothermaeota*
*Spirochaetota*	*Spirochaeta*	*Spirochaetes*
*Synergistota*	*Synergistes*	*Synergistetes*
*Thermodesulfobacteriota*	*Thermodesulfobacterium*	*Thermodesulfobacteria*
*Thermodesulfobiota*	*Thermodesulfobium*	*-*
*Thermomicrobiota*	*Thermomicrobium*	*Thermomicrobia*
*Thermoproteota*	*Thermoproteus*	*Crenarchaeota*
*Thermotogota*	*Thermotoga*	*Thermotogae*
*Verrucomicrobiota*	*Verrucomicrobium*	*Verrucomicrobia*

Derived from ref. ^7^ and other articles and validation lists published in the *International Journal of Systematic and Evolutionary Microbiology*.

Many genera were split in recent years, based on phylogenetic and phylogenomic studies. A well-known example is the renaming of *Clostridium difficile* (Hall and O’Toole 1935) Prévot 1938 (Approved Lists 1980) as *Clostridioides difficile* (Hall and O’Toole 1935) Lawson et al. 2016 gen. nov., comb. nov., creating the novel genus *Clostridioides* Lawson et al. 2016 to harbor this pathogen as it is only distantly related to the type species of the genus *Clostridium* Prazmowski 1880 (Approved Lists 1980)^8^. As the name *Clostridium difficile* was validly published, it remains validly published. Therefore, with consideration of prokaryotic nomenclature, under the rules of the ICNP, authors are free to use the older name (the basonym for the new combination *Clostridioides difficile*) if they prefer to do so^9,10^. Principle 8 and Rule 23a of the ICNP indicate that each taxon with a given circumscription, position, and rank (as defined in Principle 8 Note 2) has only one correct name. The name *Clostridioides difficile* should be used to indicate that the species belongs to the genus *Clostridioides*, as distinct from the genus *Clostridium*; the name *Clostridium difficile* should be used instead to indicate that the species belongs to the genus *Clostridium*. Only the Judicial Commission of the ICSP can reject names (Rule 56a of the ICNP). No request was yet submitted to the Judicial Commission to place the name *Clostridioides difficile* on the list of *nomina rejicienda*. Such a request can be submitted, following the procedure outlined by Article 8 of the statutes of the ICSP^11^. However, the Judicial Commission has consistently denied similar requests for rejecting genus names^12–14^.

In recent years, reclassifications have been proposed for a number of large genera, including genera of industrial or medical importance. Thus, 23 novel genera were proposed for the *Lactobacillus* group, and numerous *Lactobacillus* Beijerinck 1901 (Approved Lists 1980) species with validly published names were reclassified in these new genera^15^. Similar reclassifications of species in newly established genera were proposed for the genera *Mycobacterium* Lehmann and Neumann 1896 (Approved Lists 1980) and *Mycoplasma* Nowak 1929 (Approved Lists 1980)^16,17^. Also in these cases, all the older names that were validly published in the past can still be used by authors who prefer the older nomenclature. Following the proposed reclassification of many *Mycoplasma* species in the newly established genera *Malacoplasma*^17^, *Mesomycoplasma*^17^, *Metamycoplasma*^17^, *Mycoplasmoides*^17^, and *Mycoplasmopsis* ^17^, a Request for an Opinion was submitted to the Judicial Commission to reject the new genus names as well as the names of the newly established families *Metamycoplasmataceae* and *Mycoplasmoidaceae* and the order *Mycoplasmoidales*^18^. In Opinion 122, the Judicial Commission denied the request for a number of reasons^12^. To discuss the implications of these and other nomenclatural changes, the ICSP established an Ad hoc Committee for Mitigating Changes in Prokaryotic Nomenclature. It held its inaugural meeting in December 2023.

Most sequence- and genome databases such as the National Center for Biotechnology Information (NCBI, https://www.ncbi.nlm.nih.gov/) do not regularly publish such renamings in a transparent way, and microbiome analysis pipelines usually do not allow a “choice of nomenclature” either. Depending on analysis pipelines and databases chosen, the apparent abundance of certain taxa in metagenome results obtained for the same sample will differ, because different databases assign the same sequences to different genera.

The rules of the ICNP only apply to cultivated prokaryotes. According to Rule 30 (3) (b), as of 1 January 2001, the valid publication of the name of a new species must include the designation of a type strain, and a viable culture of that strain must be deposited in at least two publicly accessible culture collections in different countries from which subcultures must be available. To cater to the need to name uncultivated prokaryotes that can be characterized using different methods, the category ‘*Candidatus*’ was introduced in the mid-1990s^19,20^. The nomenclature of *‘Candidatus*’ taxa is not formally covered by the rules of the ICNP, and the Appendix 11 provides further explanations. More information about the correct use of the category ‘*Candidatus*’ and its limitations is found in two recent articles^21,22^. When a taxon formerly named as ‘*Candidatus*’ is cultivated, its name without the ‘*Candidatus*’ prefix can be validly published if certain conditions are met. The name must be well-formed, in accordance with the rules of the ICNP, and a viable culture of the type strain of the species on which the name of the taxon is based is available from at least two publicly accessible culture collections in different countries. An example is the valid publication of the names *Nitrososphaera* Stieglmeier et al. 2014 and *Nitrososphaera viennensis* Stieglmeier et al. 2014 in 2014, three years after the description of ‘*Candidatus* Nitrososphaera’ Tourna et al. 2011 and ‘*Candidatus* Nitrososphaera viennensis’ Tourna et al. ^23,24^. *Nitrososphaera* is the type genus of the phylum *Nitrososphaerota* Brochier-Armanet et al. 2021, formerly known as *Thaumarchaeota*. Therefore, it is important to follow the nomenclature rules of the ICNP also for ‘*Candidatus*’ taxa, so that the originally proposed names can later be validated^25^. A new proposal to emend the ICNP which would result in *Candidatus* names being regulated analogously to validly published names, was recently published^26^, and will be voted on by the ICSP in the second half of 2024.

Many uncultivated prokaroytes that can be recognized based on molecular data, from 16 S rRNA sequences to complete metagenome assembled genomes or single amplified genomes, belong to phyla that do not yet have cultivated representatives. Accordingly, a large number of ‘*Candidatus*’ phyla have been described in the literature. A curated list of 180 ‘*Candidatus*’ phyla published before the end of December 2022 was prepared, corrected in accordance with the orthography guidelines given in Appendix 9 of the ICNP and using the –*ota* ending to denote the rank of phylum. Thus, the ‘*Candidatus*’ phyla names Melainobacteriota corrig. Di Rienzi et al. 2013, Gribaldonibacteriota corrig. Probst et al. 2018, and Martarchaeota corrig. Jay et al. 2018 were proposed to replace Melainabacteria, Gribaldobacteria, and Marsarchaea, respectively^27^.

Of all the public databases that contain nomenclature information on prokaryotes, the List of Prokaryotic Names with Standing in Nomenclature database (LPSN, lpsn.dsmz.de) is recommended for information on the names of prokaryotic taxa, including their nomenclatural history and their current status: validly published or effectively published (names published in a recognized scientific printed and/or electronic publication but conditions for validation not yet fulfilled), legitimate (in accordance with the Rules of the ICNP) or illegitimate (contrary to the Rules of the ICNP), correct name, synonym, basonym (the original name of a new combination), etc^28^. It must be stressed that the only official source of information about validly published names of taxa of prokaryotes, as outlined in Rule 27 of the ICNP, is the *International Journal of Systematic and Evolutionary Microbiology*, formerly the *International Journal of Systematic Bacteriology*.

The author of this essay is happy to answer questions relating to nomenclature of prokaryotes. He can be contacted at aharon.oren@mail.huji.ac.il.
